# Bats' Conquest of a Formidable Foraging Niche: The Myriads of Nocturnally Migrating Songbirds

**DOI:** 10.1371/journal.pone.0000205

**Published:** 2007-02-14

**Authors:** Ana G. Popa-Lisseanu, Antonio Delgado-Huertas, Manuela G. Forero, Alicia Rodríguez, Raphaël Arlettaz, Carlos Ibáñez

**Affiliations:** 1 Estación Biológica de Doñana, Consejo Superior de Investigaciones Científicas (CSIC), Sevilla, Spain; 2 Estación Experimental del Zaidín, Consejo Superior de Investigaciones Científicas (CSIC), Granada, Spain; 3 Division of Conservation Biology, Zoological Institute, University of Bern, Bern, Switzerland; 4 Swiss Ornithological Institute, Valais Field Station, Nature Centre, Salgesch, Switzerland; University of Bristol, United Kingdom

## Abstract

Along food chains, i.e., at different trophic levels, the most abundant taxa often represent exceptional food reservoirs, and are hence the main target of consumers and predators. The capacity of an individual consumer to opportunistically switch towards an abundant food source, for instance, a prey that suddenly becomes available in its environment, may offer such strong selective advantages that ecological innovations may appear and spread rapidly. New predator-prey relationships are likely to evolve even faster when a diet switch involves the exploitation of an unsaturated resource for which few or no other species compete. Using stable isotopes of carbon and nitrogen as dietary tracers, we provide here strong support to the controversial hypothesis that the giant noctule bat *Nyctalus lasiopterus* feeds on the wing upon the multitude of flying passerines during their nocturnal migratory journeys, a resource which, while showing a predictable distribution in space and time, is only seasonally available. So far, no predator had been reported to exploit this extraordinarily diverse and abundant food reservoir represented by nocturnally migrating passerines.

## Introduction

According to optimal foraging theory, predators are likely to maximize their energy intake by focusing on the more abundant prey populations [1], [2]. A non-specialized predator can switch its diet towards a new prey when its growing density renders it more profitable, as a result, for instance, of a drastic environmental change or of variable resource seasonality [3]–[5]. On an evolutionary scale, the increased fitness acquired by switching to abundant, unexploited resources has been used as an explanation for adaptive radiations and speciation [6]–[8]. Numerous examples in community ecology prove that unoccupied niches still occur which offer opportunities for novel adaptations [9], [10], including the development of new foraging specializations and predator-prey interactions [11].

Bats represent an example of a tremendous adaptive radiation, probably unique in the history of mammals, that took place in the Early Eocene, coinciding with a sudden increase in plant and insect diversity and abundance [12]. In the course of a few million years, bats colonized most ecological niches and learnt to exploit a wide array of food sources including arthropods, pollen, fruit, small terrestrial vertebrates and even blood [13]. They have co-evolved with the species that constitute their diet [14], sometimes resulting in fascinating predator-prey interactions such as the avoidance and counter-avoidance strategies developed between some echolocating aerial-hawking bats and their tympanate prey (the “allotonic frequency hypothesis”, e.g. [15]).

**Figure 1:**
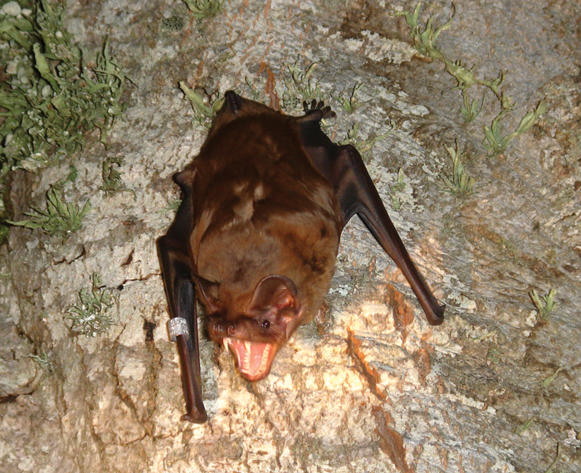
*Nyctalus lasiopterus* showing its impressive teeth to the researchers.

The unexpected finding of bird feathers in the faecal pellets of *Nyctalus lasiopterus* [16], [17] (Figure 1), a rare Mediterranean aerial-hawking bat, caused substantial controversy. The conclusion drawn by the authors that giant noctules feed on migrating birds [17] was severely criticized by some scientists who suggested a mistaken or selective ingestion of hovering feathers but not birds [18]. The reason for this scepticism was probably the discovery of a previously undocumented foraging strategy and predator-prey relationship: indeed, no predators were known to hunt in the open upon nocturnally migrating birds, despite the immense resource that the latter potentially represent. Owing to this disbelief, a technique was needed which would identify not only ingested, but also assimilated food. We therefore relied on stable isotope analysis, a method that is becoming increasingly popular in trophic ecology for discriminating between potential food sources, documenting temporal changes in diet and estimating trophic level [3], [19].

Using stable isotopes of carbon and nitrogen, we challenged the hypothesis of carnivory in the giant noctule bat. Comparison of isotopic values of a specific tissue (e.g. blood, hairs, feathers, etc.) over time is considered the most straightforward method for assessing temporal variation in diet [20]. The choice of a specific tissue will enable diet changes to be recorded at the desired time scale depending on that tissue's metabolic turnover [20]. For our purposes, whole blood seemed the most appropriate tissue to track seasonal dietary patterns, given its relatively rapid turnover integrating short-term dietary variation in terms of days or weeks [20]–[22]. Carbon and nitrogen half-lives in the blood of small mammals are in the order of 15–30 days [23], [24], but the change in isotopic composition of a consumer after a major diet switch through time follows an exponential model, so that dietary changes could be reflected in a consumer's tissue within a day [25]–[27]. In fact, Mirón *et al.* [24] showed that whole blood is a suitable tissue to track seasonal dietary shifts in bats.

Accordingly, we explored seasonal variation of stable isotopes in the blood of *Nyctalus lasiopterus* and compared it with the isotopic signatures of its potential prey: insects and birds. We predicted that the temporal variability of stable isotope ratios would reflect an insect-based diet in summer, and incorporation of birds into the diet in spring and autumn during passerine migration, with a major contrast between summer, when diet is mostly based on insects, and autumn, when the mass occurrence of migratory songbirds would induce a prominent dietary bias towards vertebrate flesh [17]. A confirmation of this chronological pattern would provide solid evidence of predation upon birds by giant noctule bats.

## Results

### Isotopic signatures of potential prey

Overall mean (±SE) isotopic values of passerines (δ^13^C: −22.43±0.32‰; δ^15^N: 9.60±0.26‰; *n* = 29) and invertebrates (δ^13^C: −25.64±0.23‰; δ^15^N: 6.45±0.39‰; *n* = 80) differed significantly for combined isotopes (MANOVA: Wilks' lambda = 0.62, *F*_2,106_ = 32.95, *P*<0.001) and for each isotope separately (ANOVA: δ^13^C: *F*_1,107_ = 56.09, *P*<0.001; δ^15^N: *F*_1,107_ = 22.46, *P*<0.001).

**Figure 2:**
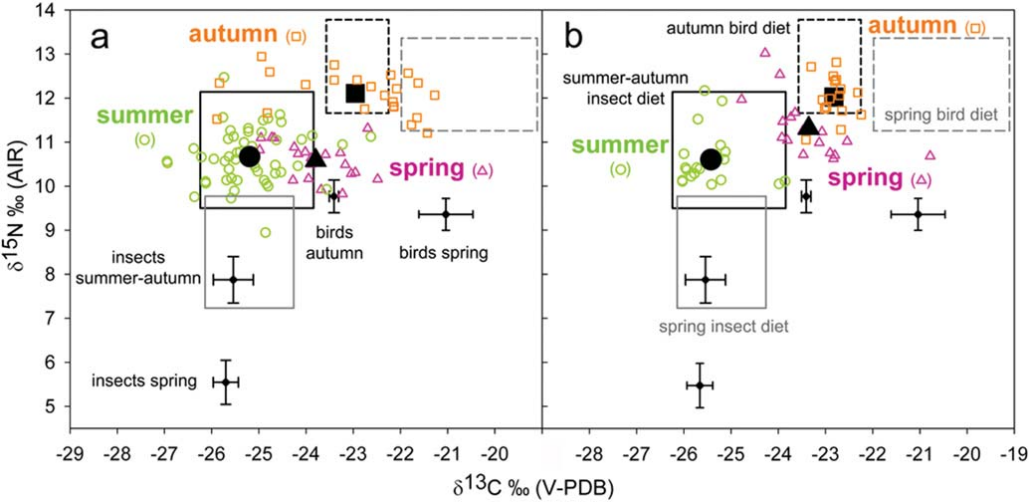
Stable isotope values (δ^13^C and δ^15^N) found in bat blood in 2003 (a) and 2004 (b). Individual samples are depicted by small, empty symbols in color, whilst large black symbols show corresponding average group values (seasons are shown with different colours; labelling on both graphs). Mean (±SE) values for potential prey are also shown as small diamonds with bi-directional error bars (labels in 2a). Boxes represent the range of expected values, considering 99% CI for prey and accounting for fractionation factors in the blood of bats that would mirror a pure invertebrate diet (full frames) or a pure bird diet (dashed frames) in spring (grey frames), summer and autumn (black frames, labels in 2b), respectively (see Methods). Partial labelling for prey isotopic signatures («crosses») and expected dietary isotopic signatures (rectangular frames) are shown to enhance clarity.

When looking at seasonal variation, it appeared that invertebrates in spring (δ^13^C: −25.70±0.27‰; δ^15^N: 5.55±0.50‰; *n* = 49; the lower «cross» in Figure 2) and in summer-autumn (δ^13^C: −25.54±0.42‰; δ^15^N: 7.87±0.53‰; *n* = 31; the «cross» just above the former in Figure 2) differed significantly (MANOVA: Wilks' lambda = 0.89, *F*_2,77_ = 4.86, *P* = 0.01), with summer-autumn samples being significantly enriched in ^15^N (ANOVA δ^15^N: *F*_1,78_ = 9.46, *P* = 0.0030), whereas δ^13^C did not differ between spring and summer-autumn (ANOVA δ^13^C: *F*_1,78_ = 0.11, *P* = 0.74).

Migrating passerines returning from their wintering grounds in spring (δ^13^C: −21.04±0.57‰; δ^15^N: 9.36±0.36‰; *n* = 12; rightmost «cross» in Figure 2) and passerines leaving their breeding areas in autumn (δ^13^C: −23.41±0.10‰; δ^15^N: 9.77±0.37‰; *n* = 17; second «cross» from the right, Figure 2) differed significantly in isotopic signatures (MANOVA: Wilks' lambda = 0.51, *F*_2,26_ = 12.32, *P*<0.001), the former being enriched in ^13^C (ANOVA δ^13^C: *F*_1,27_ = 23.27, *P*<0.001), while both groups showed similar δ^15^N values (ANOVA δ^15^N: *F*_1,27_ = 0.58, *P* = 0.45).

Invertebrates in summer-autumn and migrating passerines in autumn were clearly distinguishable (MANOVA: Wilks' lambda = 0.73, *F*_2,45_ = 8.14, *P*<0.001; ANOVA, δ^13^C: *F*_1,46_ = 13.60, *P*<0.001; δ^15^N: *F*_1,46_ = 6.12, *P* = 0.017). As a result, we can expect a marked isotopic signature contrast between a mostly invertebrate-based diet in summer-autumn and a pure-bird diet in the same season.

### Isotopic signatures of bat blood

For blood samples collected in 2003, we found first a significant overall effect of season on the signatures of the two isotopes (MANOVA: Wilks' lambda = 0.25, *F*_4,178_ = 44.02, *P*<0.001). Values also differed seasonally for each isotope taken separately (one-way ANOVA; δ^13^C: *F*_2,90_ = 43.24, *P*<0.001; δ^15^N: *F*_2,90_ = 61.85, *P*<0.001). Post-hoc pairwise comparisons further showed that all three seasons segregated from one another in their δ^13^C values, with autumn having the highest mean value followed by spring. For δ^15^N, autumn differed significantly from the other two seasons, which both exhibited lower average values (Figure 2A). In 2004, the pattern was very similar, showing also significant seasonal differences (MANOVA: Wilks' lambda = 0.13, *F*_4,102_ = 46.49, *P*<0.001; one-way ANOVA: δ^13^C: *F*_2,52_ = 91.66, *P*<0.001; δ^15^N: *F*_2,52_ = 27.21, *P*<0.001). Scheffé post-hoc comparisons showed that all pairs of seasons were significantly different from each other in δ^15^N; autumn exhibited the highest average values followed firstly by spring and secondly by summer. In terms of δ^13^C, only summer was significantly different from the other seasons (Figure 2B).

### Expected composition of consumers exploiting a single prey source

The range of estimated δ^13^C and δ^15^N values for bats that would feed only upon invertebrates vs solely on birds, with respect to season, are presented as rectangles in Figure 2. There was no overlap between expected values for an invertebrate diet and for a bird diet, independently of the season. Presumed bird diets in spring and in autumn also showed no overlap; presumed invertebrate diets for different seasons overlapped slightly.

## Discussion

To test the hypothesis of passerine predation by giant noctule bats during bird migration events, for each season we built theoretical ranges in which the isotopic composition in the blood of bats feeding on a pure invertebrate or on a pure bird diet should lie (Figure 2). Prey categories were significantly different from each other isotopically: passerines had higher values than invertebrates, what corresponds to their superior position in the trophic chain. The two invertebrate groups were analogous in terms of δ^13^C, but δ^15^N was higher for invertebrates in the marshland, where giant noctules forage during summer and autumn. Regarding the bird resource (Table 1), the enrichment in δ^13^C of spring migrants returning from their African winterquarters relative to autumn migrants was consistent with the acknowledged existence of a latitudinal gradient in δ^13^C [28]. The resulting boxes encompassing predicted values did not overlap, except for both categories of predicted invertebrate diet. The expected values for an invertebrate diet in summer-autumn and for a bird diet in autumn showed no overlap (Figure 2); isotopic differentiation of these two prey categories is crucial for identifying any hypothetic diet switch in the bats between summer and autumn.

**Table 1:** Bird samples analysed to predict isotopic signatures of *Nyctalus lasiopterus'* diet.

Season	Species	*n*
Spring	*Delichon urbica*	10
	*Sylvia cantillans*	2
Autumn	*Erithacus rubecula*	4
	*Phoenicurus ochruros*	3
	*Phylloscopus* sp.	1
	*Phylloscopus collybita*	4
	*Phylloscopus ibericus*	1
	*Phylloscopus trochillus*	1
	*Sylvia atricapilla*	2
	*Sylvia melanocephala*	1

Bird samples analysed to predict isotopic signatures of *Nyctalus lasiopterus*' diet with respect to season of collection (spring: pre-nuptial migration; autumn: post-nuptial migration, from late summer to early winter) and bird species.

When plotting the bat blood isotopic data together with the ranges of expected values, summer (characterized by the absence of bird migration in the study area) was, both for 2003 and 2004, the only season for which blood composition fell inside the predicted pure invertebrate diet (Figure 2). Blood isotopic data for autumn fell in the lower part of the box representing a theoretical pure-bird diet. This strongly suggests that passerines are the main source of assimilated nutrients for the bats during this season, although in terms of ingested diet, feathers accounted for only about 50% of faecal pellets content [17]. In spring, blood isotopic composition exhibited intermediate values between those of summer and autumn, falling between the expected predicted values for a pure-invertebrate and a pure-bird diet. These results support a moderate contribution of birds to the diet of giant noctules in spring and a pronounced diet shift from invertebrates to birds from summer to autumn. During spring, concentrations of migratory birds in Southern Spain are lower than in autumn [29], as winter mortality renders the spring migratory flux less massive. In addition, autumn is characterized by the occurrence of young naïve birds conducting their first migration, i.e. possibly more vulnerable to predation than experienced individuals.

While average values for both summer and autumn were very consistent between years, spring exhibited considerable variability. For 2004, when seasonal captures were conducted in single sessions rendering comparisons between them meaningful, spring was also the season in which dispersion of the data was greatest. Bird availability in spring is lower than in autumn; as a consequence, passerines should be harder to find and capture. We could thus expect more variability in bat diet composition, i.e. higher variance in the isotopic signatures of bats in spring, what we actually observed. Indeed, isotopic variance among individuals has been suggested as a reliable indicator of omnivory or opportunistic feeding [30].

**Figure 3:**
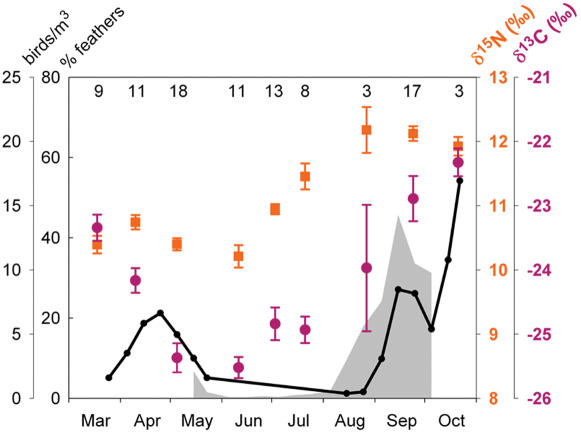
Seasonal variation in isotopic composition of blood of *Nyctalus lasiopterus.* Means±SE for δ^13^C (purple circles) and δ^15^N (orange squares) for each sampling event are given. Numbers at the top of the figure designate sample size. The black line indicates nocturnal migration density of small passerines tracked by radar in southern Spain [29]. The shaded area represents proportion of feathers found in faecal pellets of *Nyctalus lasiopterus* [17].

The seasonal pattern of variation of both isotopes shows a remarkable correspondence with the passerine migration density curve recorded through radar observations in southern Spain and with the annual variation in the proportion of feathers found in faeces (Figure 3). Although the timing of migration can vary slightly (1–2 weeks shift) between years [31], the density of migration in the study area follows a highly predictible chronological pattern within a year cycle. Both isotopes experience a first and a second spike which coincide with the two major migration peaks in spring and autumn, respectively. The early rise in δ^15^N through June and July, when no bird migration takes place, is probably caused by the observed change in foraging habits of the bats (Popa-Lisseanu & Ibáñez, unpublished) exploiting primarily the more ^15^N enriched invertebrates of the marshlands.

Our results strongly support a seasonal carnivory in *Nyctalus lasiopterus*, in contrast to other alternative hypotheses, such as a mistaken ingestion of feathers [18]. The almost perfect match of the blood isotopic composition to a pure-invertebrate diet in summer, a period with no migration, provides further support against the view that the bats casually catch birds in tree cavities or nestboxes while visiting potential roosts [16]; indeed, many small passerines occupy tree cavities and nestboxes during reproduction in spring and early summer in the study area.

While the ability to capture vertebrate prey is well documented in tropical bats that use a gleaning foraging strategy [32], the morphology and echolocation characteristics of *Nyctalus lasiopterus* are adapted to a strictly aerial-hawking strategy [17], [33], with a poor ability to manoeuvre in close vegetation and in the presence of obstacles. Moreover, like other members of the tribe Pipistrellini (Vespertiolinidae), giant noctules never take their prey to perches [34]. Instead, they consume them on the wing, typically discarding the non-profitable parts. This implies that giant noctules are the only recognized nocturnal predators capable of feeding upon birds in the air. Although the exact way that bats subdue their prey remains a mystery, we presume that the great elevation at which passerines are known to migrate [35] allows giant noctules to capture, prepare and ingest birds in the air without facing any risk of collision with obstacles.

Every year, approximately five billion passerines cross the Mediterranean basin during their autumn migrations [36]. A big proportion of them are small-sized; as an example, more than 90% of migrating passerines mist-netted in the study area have an average body mass of less than 20 g (data from the 2004 ringing campaign of migratory passerines in the Doñana National Park, Equipo de Seguimiento de Procesos Naturales, Estación Biológica de Doñana). They thus represent a multitude of potential hunting targets for a large bat like *Nyctalus lasiopterus* [17]. The presence in its habitat of such an abundant, high-quality food resource during large parts of the yearly activity cycle would provide the potential for such high fitness advantages that it is in the end not very surprising that giant noctules opportunistically switch from an insectivorous diet to a partly carnivorous diet at some periods of the year. Furthermore, the bats would not have to compete against other potential predators, none of which are known to exploit that extraordinary food source. In this respect, the question arises to what extent such a big bat as *Nyctalus lasiopterus* actually depends on this resource for its survival. On one hand, there is echolocation which constrains the upper size of aerial-hawking bats feeding on insects [37]. On the other hand, the distribution of this rare species seems almost exclusively Mediterranean [38], which suggests that its geographical distribution may be restricted to areas where the main migration streams of Palaearctic passerines occur [29]. Giant noctule bats could be subject to similar selective pressures and show ecological convergences with Eleonora's falcon (*Falco eleonorae*). This diurnal raptor breeds in the fall – which contrasts with all other western Palaearctic bird species - preying upon migratory passerines that fly along coastlines and cross the Mediterranean Sea and the Red Sea on their way to Africa [39]. Most of its colonies are therefore on islands and sheer coastal cliffs along major autumn migration routes.

Through the use of modern stable isotope techniques, we provide here new evidence for predation of giant noctule bats upon nocturnally migrating passerines. Elucidating the mechanics of this hunting strategy, as well as details of this predator-prey interaction, will be especially challenging given that most nocturnal passerine migration takes place at great elevations [29]. The question remains whether other aerial-hawking bat species may have developed similar foraging skills, enabling them to exploit the outstanding food reservoir represented by nocturnally migrating birds.

## Materials and Methods

### Study area and collection of samples

Bats were mist-netted monthly at two maternity colonies located in Seville and in the Doñana National Park (Andalusia, Spain) during a full activity cycle (from March until end of October) in 2003. A few microlitres of blood were extracted from the caudal vein in the interfemoral membrane [40]. Only samples from adult females were used to avoid sex and age-specific biases. Tissues from potential prey were also collected. Caught with Malaise and light traps at night during summer and autumn 2003 and spring 2004, arthropod samples comprised in total ten insect orders plus some specimens of Araneae and Opiliones, representing all taxa found in faecal pellets of *Nyctalus lasiopterus* [16]. Our reference sample for birds included muscle from 29 small migratory passerines which died accidentally during ringing campaigns in Andalusia in the period 1996–2006. All samples were preserved at room temperature in 70% ethanol [41]. To check for between-year consistency of the results, we sampled again in 2004, following the same protocols, the bat colony of Seville, during three 3-day capture sessions, in April, June–July and September–October, respectively. Capture and handling of the bats were carried out under license of the Junta de Andalucía.

### Stable isotope analysis

Isotope measurements were conducted at the Stable Isotope Laboratory of the Estación Experimental del Zaidín (CSIC, Granada, Spain). Ethanol was removed from all samples prior to analysis by freeze-drying. Samples (about 0.5–1 mg) were combusted at 1020°C using a continuous-flow system by means of an EA-IRMS elemental analyzer (Carlo Erba 1500NC) on line with a Delta Plus XL mass spectrometer. Helium was used as the carrier gas. The stable composition was reported as δ values per mil (‰) using the formula:
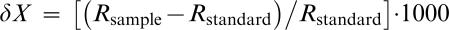
where *X* is either ^13^C or ^15^N, and *R* the proportion ^13^C/^12^C or ^15^N/^14^N ratios. The original standard reference for carbon is PDB (Pee Dee Belemnites, a fossil marine carbonate of biogenic origin) and for nitrogen (AIR) an average of ^15^N/^14^N from atmospheric air.

All samples were analysed by duplicate on different days. Usually two standards were measured every ten samples. The overall precision of analyses was ±0.1‰ for both δ^13^C and δ^15^N.

### Statistical analysis

Assessment of temporal variation in the diet of a consumer through stable isotopes requires that the possible diet sources have distinct isotopic signatures. Additionally, isotopic variation of the dietary sources themselves could cause a temporal pattern in the isotopic composition of a consumer even if it has not changed its diet [20], resulting in erroneous interpretations. We thus considered possible variation in the signatures of potential prey. For obtaining a better estimate of the invertebrates consumed by the bats in each season, we took into account the seasonal foraging habits of *Nyctalus lasiopterus*: whereas in spring the bats foraged in diverse habitats not far from Seville, in summer and in autumn they travelled long distances to forage in the marshlands of the Guadalquivir river south of Seville (Popa-Lisseanu & Ibáñez, unpublished). Invertebrates were thus divided into two categories: invertebrates available in spring, comprising specimens trapped in different ecosystems near Seville, and invertebrates available in summer and autumn, most of them collected near the Doñana marshlands (Table 2). To comply with possible seasonal variation in the avian resource as a result of distinct isotopic signatures of migratory birds coming either from Europe or from Africa, passerines were also grouped into two categories (Table 1): migrants captured in spring (arriving from Africa), and migrants captured in autumn-winter (arriving from Europe). To assess seasonal variation in the blood of the bats in both 2003 and 2004, three seasons were distinguished, based on temporal patterns of passerine migration phenology in the Doñana National Park: prenuptial or spring migration (March–April), reproductive season with absence of migration (May–July), and postnuptial or autumn migration (August–October), the latter being characterized by mass flights of songbirds across southern Spain.

**Table 2:** Invertebrate samples analysed to predict isotopic signatures in the diet of *Nyctalus lasiopterus*.

Season	Habitat	Taxon	*n*
Spring	Mediterranean forest	Araneae	1
		Coleoptera	5
		Diptera	5
		Hymenoptera	3
		Lepidoptera	6
		Orthoptera	1
		Trychoptera	2
	Scrubland	Araneae	2
		Coleoptera	2
		Diptera	1
		Heteroptera	1
		Neuroptera	1
		Opiliones	1
		Orthoptera	3
	Wooded pasture (“dehesa”)	Heteroptera	2
		Homoptera	4
		Lepidoptera	6
	Riverine thicket	Opiliones	1
		Trychoptera	1
	Urban	Heteroptera	1
Summer-autumn	Dunar pinewood in marshland	Coleoptera	11
		Diptera	1
		Heteroptera	2
		Lepidoptera	6
		Neuroptera	1
	Marshes	Coleoptera	2
	Pasture in marshland	Coleoptera	2
	Urban	Diptera	1
		Heteroptera	1
		Hymenoptera	1
		Odonata	2
		Orthoptera	1

Invertebrate samples analysed to predict isotopic signatures in the diet of *Nyctalus lasiopterus* with respect to habitat type and taxa. To account for seasonal variation, samples were grouped into two categories according to the seasonal foraging behaviour of the bats (Popa-Lisseanu & Ibáñez, unpublished).

For both carbon and nitrogen, differences in isotopic signatures between the two types of prey, consumer and prey, and among seasons within each analysed group were tested first for the two isotopes combined (MANOVA), then for carbon and nitrogen taken separately (one-way ANOVA), with Scheffé post-hoc comparisons. Values are expressed as means±SE. The statistical software used was SPSS version 12.0.

### Calculation of expected isotopic signatures in consumers

Based on the isotopic signatures of the potential prey (means±99% confidence intervals), we predicted for each season the range of expected isotopic values to be found in the blood of the bats in the case of a pure invertebrate diet and for a diet based entirely on birds. Intermediate diets would presumably lie somewhere in between, depending first on the proportion of ingestion of each of these two main prey categories, and second on their respective efficiency of assimilation and specific nutrient routings [42]. When a diet is incorporated into the consumer's tissues, a predictable step change, or fractionation, in its isotopic composition occurs, caused by preferential retention of heavier isotopes in the body [43], [44]. For our predictions, we used typical fractionation ranges found in vertebrates, namely 0–1‰ for δ^13^C and 2.5–3.5‰ for δ^15^N [22], [45]–[47]. Our choice is justified by the recent recognition that discrimination factors in the blood of nectarivorous bats fed on a good quality diet fall within this range (0.1‰ and 3.3‰, for δ^13^C and δ^15^N, respectively [24]). Unfortunately, no similar experiments based on a controlled diet have been conducted so far with captive insectivorous bats. The pattern of monthly isotopic variation in the blood of the bats sampled in 2003 was compared with the migration density curve of small passerines tracked by radar in Vélez-Málaga in 1996 [29], about 150 km from the study area, and with the annual variation in the proportion of feathers found in faeces of *Nyctalus lasiopterus* in Jerez de la Frontera, southern Spain, in 2001 [17].
